# Temozolomide–perillyl alcohol conjugate downregulates O^6^-methylguanin DNA methltransferase via inducing ubiquitination-dependent proteolysis in non-small cell lung cancer

**DOI:** 10.1038/s41419-017-0193-2

**Published:** 2018-02-09

**Authors:** Xingguo Song, Li Xie, Minghui Chang, Xinran Geng, Xingwu Wang, Thomas C. Chen, Xianrang Song

**Affiliations:** 1 Shandong Provincial Key Laboratory of Radiation Oncology, Shandong Cancer Hospital Affiliated to Shandong University, Shandong Academy of Medical Sciences, Jinan, Shandong China; 2 Department of Clinical Laboratory, Shandong Cancer Hospital Affiliated to Shandong University, Shandong Academy of Medical Sciences, Jinan, Shandong China; 3 School of Medicine and Life Sciences, University of Jinan, Shandong Academy of Medicine Science, Jinan, Shandong China; 4 Maternity & Child Care Center of Dezhou, Dongdizhong Street 835#, Decheng District, Dezhou, Shandong China; 5 Departments of Neurological Surgery and Pathology, University of Southern California, Los Angeles, CA USA

## Abstract

The DNA repair enzyme O^6^-methylguanin-DNA-methltransferase (MGMT) is able to remove products of alkylating agent such as O^6^-meG and emerges as a central determinant of cancer resistance to temozolomide (TMZ). Temozolomide–perillyl alcohol conjugate (TMZ–POH), a novel TMZ analog developed based on the conjugation of TMZ and POH, displayed strong anticancer potency in multiple cancer types, but seemed not to experience the chemoresistance even in cells with high MGMT expression unlike TMZ and other alkylating agents. In this study, we demonstrated TMZ–POH inhibited MGMT dependent on proteasomal pathway and this inhibition is a significant factor in its toxic effect in the non-small cell lung cancer (NSCLC) cells.

## Introduction

Nowadays, chemotherapy has been traditionally considered as one of the standard treatment options for cancer patients, but the chemoresistance dramatically hinders its clinical application, especially for alkylating agents like temozolomide (TMZ)^1^. TMZ, an imidazotetrazine derivative of the alkylating agent dacarbazine, was approved by the US FDA (Food and Drug Administration) and European Union^2^. Its cytotoxicity is mainly due to its capability to react with DNA to form methyl adducts^3^, such as O6-methyl-guanine (O^6^-meG), which trigger cell cycle-dependent DNA damage and cell death^4^. Although TMZ chemotherapy may enhance survival of cancer patients, intrinsic or acquired resistance to TMZ is also common and accounts for many treatment failures^5^, because TMZ-induced DNA alkylation damage can be repaired by O^6^-methylguanin-DNA-methltransferase (MGMT)^6^.

MGMT, an evolutionary conserved enzyme whose high expression indicates poor prognosis in multiple cancers^7^, plays a crucial role in the defense against alkylating agents^8^. MGMT is able to remove O^6^-meG which covalently is attached to the protein and inactivates it^9^, and emerges as a central determinant of tumor resistance to alkylating agents. Ongoing studies have intended to inhibit MGMT activity to enhance the therapeutic effect of TMZ^10^. Despite the development and application of many MGMT inhibitors including O^6^-benzylguanine (O^6^-BG)^11^, lomeguatrib^12^, and their corresponding derivatives, the efficacy of MGMT inhibitors combined with alkylating agents remains controversial due to their hematologic toxicity and inefficiency^13–15^.

TMZ–perillyl alcohol conjugate (TMZ–POH), a novel TMZ analog^16^, is developed based on the conjugation of TMZ and POH, the latter is a naturally occurring monoterpene which has the amazing capability to enhance the cytotoxicity of TMZ in several tumors^17^. Previous studies had revealed that TMZ–POH displayed stronger anticancer potency than its individual constituents to several types of malignancy such as triple-negative breast cancer (TNBC)^16^, non-small cell lung cancer (NSCLC)^18^, human nasopharyngeal carcinoma (NPC)^19^, and even TMZ-resistant gliomas^20^. Unlike TMZ and other alkylating agents, TMZ–POH seems not to experience the chemoresistance even in cells with high MGMT expression. To illuminate this issue, in this study we aimed to explore the mechanisms of TMZ–POH-regulating MGMT.

Accumulating evidences prove that MGMT is regulated by multiple mechanisms. MGMT is epigenetically silenced by its promoter methylation in many cancer types^21,22^, and its expression is regulated by Wnt/β-catenin signaling as the direct transcriptional target of β-catenin^7^. Besides, MGMT protein can be modified by multiple pathways. In mammals, it is a short-lived protein, degraded via ubiquitination-dependent proteolysis^23^, and acts as a proteolytic target for the E6 human papilloma virus oncoprotein^24^. In yeast, MGMT is a physiological substrate of both the Ubr1-dependent N-end rule pathway and the Ufd4-dependent Ub fusion degradation (UFD) pathway^25^.

Given the importance and complicated regulation of MGMT, in this paper we focused on the role of TMZ–POH in MGMT regulation, and that of MGMT in TMZ–POH’s cytotoxicity in multiple cancer cells. We show that TMZ–POH inhibits MGMT dependent on proteasomal pathway and this inhibition is a significant factor in its toxic effect in NSCLC cells, thus proposing TMZ–POH as a potential therapeutic candidate for NSCLC.

## Results

### TMZ–POH downregulates MGMT protein in a concentration and time-dependent manner

**Fig. 1:**
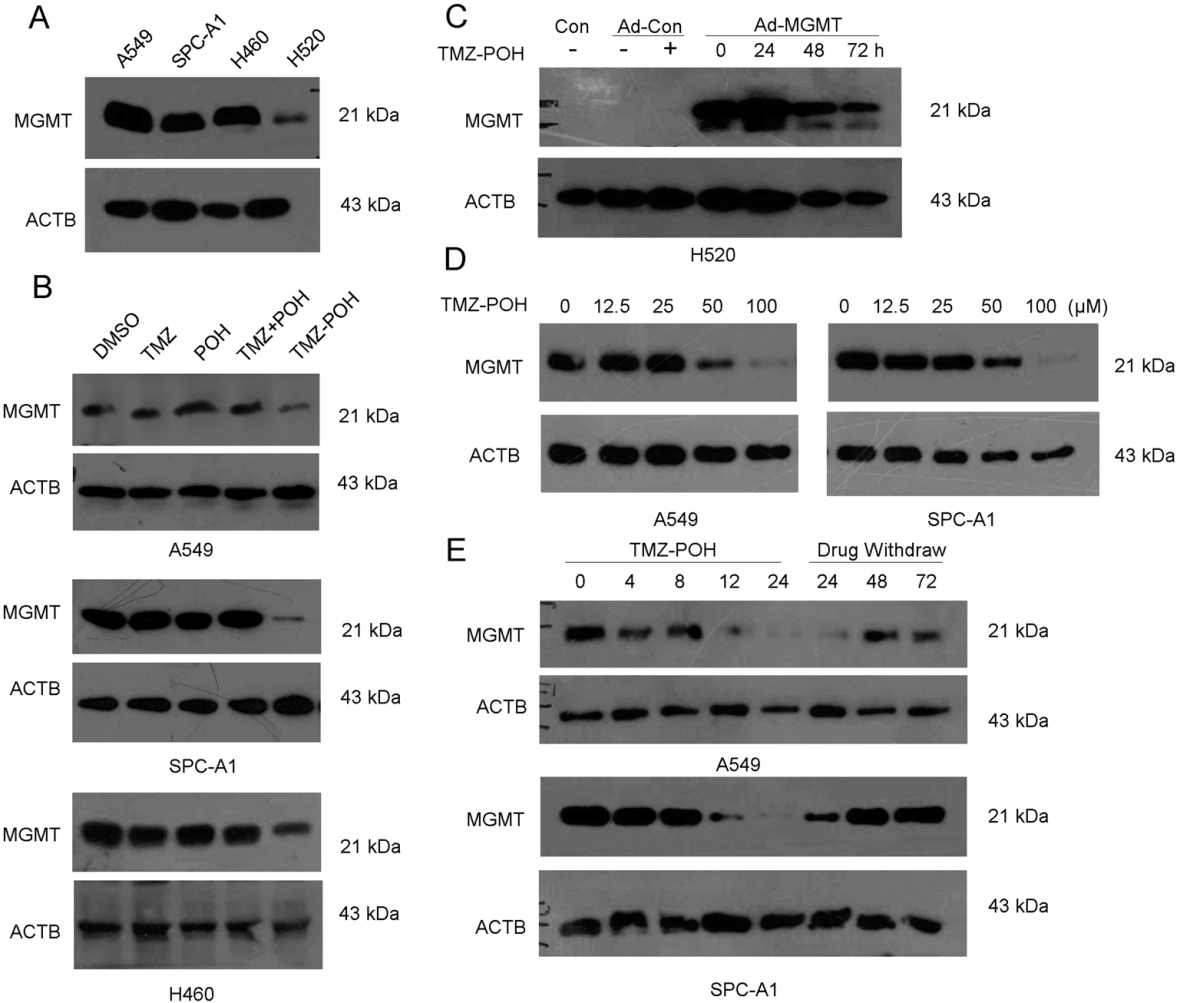
TMZ–POH downregulates MGMT protein in a concentration-dependent and time-dependent manner. **a** MGMT protein level was evaluated by western blots in A549, SPC-A1, H460, and H520 cells. **b** MGMT protein level was detected in A549, SPC-A1, and H460 cells following treatment with 100 μM TMZ, POH, TMZ + POH (100 μM TMZ + 100 μM POH), TMZ–POH, or DMSO for 48 h, respectively. **c** H520 cells were infected by Ad-MGMT or control for 24 h followed by 100 μM TMZ–POH treatment for indicated time. MGMT protein level was detected by western blots. **d** MGMT protein level was detected in A549, SPC-A1 cells following treatment with 0, 12.5, 25, 50, 100 μM TMZ–POH for 48 h, respectively. **e** MGMT protein level was detected in A549, SPC-A1 cells treated with 100 μM TMZ–POH for 0, 4, 8, 12, 24 h, followed by washing three times with PBS and incubation in drug-free media for next 24, 48, 72 h, respectively

In order to illuminate the effect of TMZ–POH on MGMT protein, we detected MGMT protein expression in four different NSCLC cell, including A549, SPC-A1, NCI-H460, and NCI-H520. As shown Fig. 1a, A549, SPC-A1, and NCI-H460 expressed high level of MGMT protein whereas H520 expressed low. Next, endogenous MGMT protein level followed by 100 μM TMZ, POH, TMZ + POH (100 μM TMZ plus 100 μM POH), TMZ–POH or DMSO for 48 h, respectively, was detected in A549, SPC-A1, and H460 cells. TMZ–POH treatment resulted in endogenous MGMT protein downregulation compared to its individual constituents and their combination (Fig. 1b). Moreover, H520 was employed and subject to infection of recombinant adenoviral vector carrying the human MGMT gene (Ad-MGMT). Although adenoviral infection led to sustained MGMT expression, TMZ–POH downregulated the exogenous MGMT protein after 48 and 72 h of treatment significantly (Fig. 1c). In addition, the phenomenon TMZ–POH-inhibited MGMT protein expression was also observed in other cancer-derived cells, including ovarian carcinoma-derived cell A2780, human NPC-derived cell lines CNE2, and glioma-derived cell line T98G as shown in Figure S1. These results suggest TMZ–POH inhibited both endogenous and exogenous MGMT protein, independent of cell type.

Besides, A549 and SPC-A1 cells were exposed to TMZ–POH with different concentrations, and MGMT protein level was detected. As shown in Fig. 1d, MGMT protein was not affected by low concentration (12.5 and 25 μM) of TMZ–POH, but suppressed significantly by high concentration (50 and 100 μM) TMZ–POH, indicating TMZ–POH-downregulated MGMT dependent on concentration. In addition, we delineated the appropriate time for TMZ–POH treatments following MGMT downregulation. The presence of 100 µM TMZ–POH caused a significant decrease of MGMT protein from 12 h until 24 h in A549 and SPC-A1 cells (Fig. 1e). It has been reported that MGMT performs a stoichiometric reaction to accomplish the DNA repair and is inactivated and degraded, it is not recycled to its active form but restored due to fresh translation in cells^26,27^. Therefore, TMZ–POH was withdrawn to allow MGMT regeneration for another 24, 48, and 72 h after incubation for 24 h, and then MGMT protein levels were analyzed by western blot. As expected, MGMT protein level was restored when TMZ–POH was evacuated (Fig. 1e), suggesting TMZ–POH functioned as a transient inhibitor of MGMT. Taken together, our data support TMZ–POH downregulates MGMT in a concentration and time-dependent manner.

### MGMT downregulation is required for TMZ–POH’s cytotoxicity

**Fig. 2:**
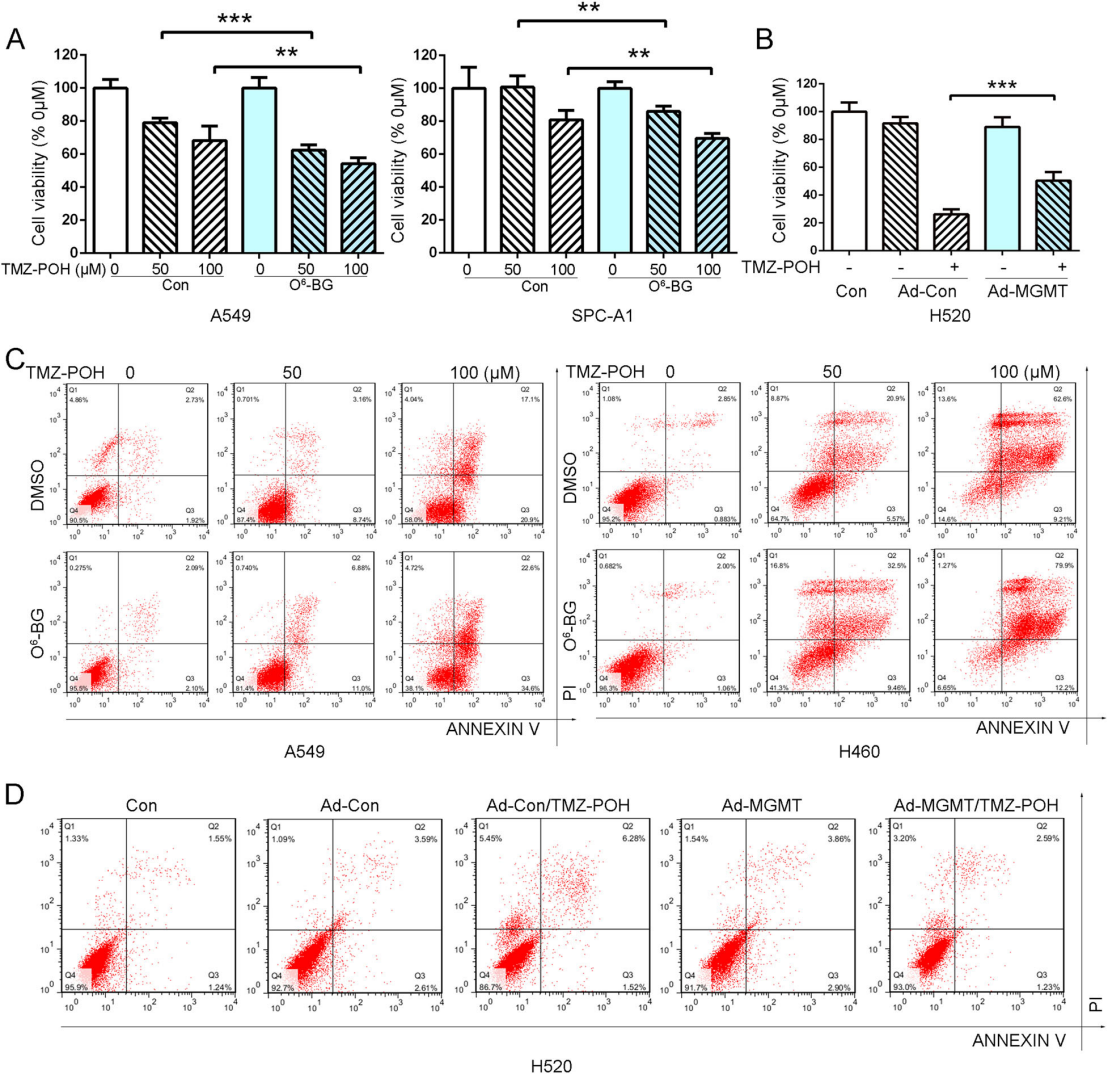
MGMT downregulation is required for TMZ–POH’s cytotoxicity. **a** A549 and SPC-A1 cells were treated with 0, 50, 100 μM TMZ–POH for 48 h with or without presence of 25 μM O^6^-BG, respectively, and then subjected to MTT assay, DMSO acted as the control. Absorbance value was calculated and standardized to DMSO group. **b** H520 cells was infected by Ad-MGMT or control for 24 h followed by 100 μM TMZ–POH treatment for 48 h. Cell viability was detected by MTT assay. **c** A549 and H460 cells were treated with 0, 50, 100 μM TMZ–POH for 48 h with or without presence of 25 μM O^6^-BG, respectively, and then subjected to apoptosis assay. **d** H520 cells was infected by Ad-MGMT or control for 24 h followed by 100 μM TMZ–POH treatment for 48 h, and then subjected to apoptosis assay. The results shown are means ± SD; **p < 0.01; ***p < 0.001

Our previous study has revealed that TMZ–POH exhibited its cytotoxic effect on NSCLC^18^. To explore the influence of MGMT on TMZ–POH’s cytotoxicity, O^6^-benzylguanine (O^6^-BG), an established inhibitor of MGMT and currently undergoing clinical trials^15^, was administrated and combined with TMZ–POH. Cell viability was assayed by the MT assay. As shown in Fig. 2a, combination of TMZ–POH (50 and 100 μM) and O^6^-BG (25 μM) suppressed cell proliferation in A549 and SPC-A1 cells. Nevertheless, another NSCLC cell line H520 was employed and subject to infection of Ad-MGMT, which significantly alleviated TMZ–POH’s cytotoxicity (Fig. 2b).

To determine whether MGMT regulated TMZ–POH-induced cell death, apoptosis detection assay was also performed when treated with TMZ–POH in the presence or absence of MGMT expression. As shown in Fig. 2c, flow cytometry analysis displayed that O^6^-BG facilitated TMZ–POH-induced cell death as evidence from much more apoptotic or dead cells when treated with their combination in A549 and H460 cells for 48 h, whereas MGMT overexpression by adenoviral infection prevented TMZ–POH from inducing cell death in H520 cells (Fig. 2d). Otherwise, TMZ alone exerted no obvious effect on apoptosis in A549 cells, but more apoptotic or dead cells were detected when combined with O^6^-BG (Fig. S2), consistent with the previous review that O^6^-BG overcame TMZ resistance ensued by MGMT^14^.

### MGMT downregulation is required for cell cycle-dependent DNA damage induced by TMZ–POH

**Fig. 3:**
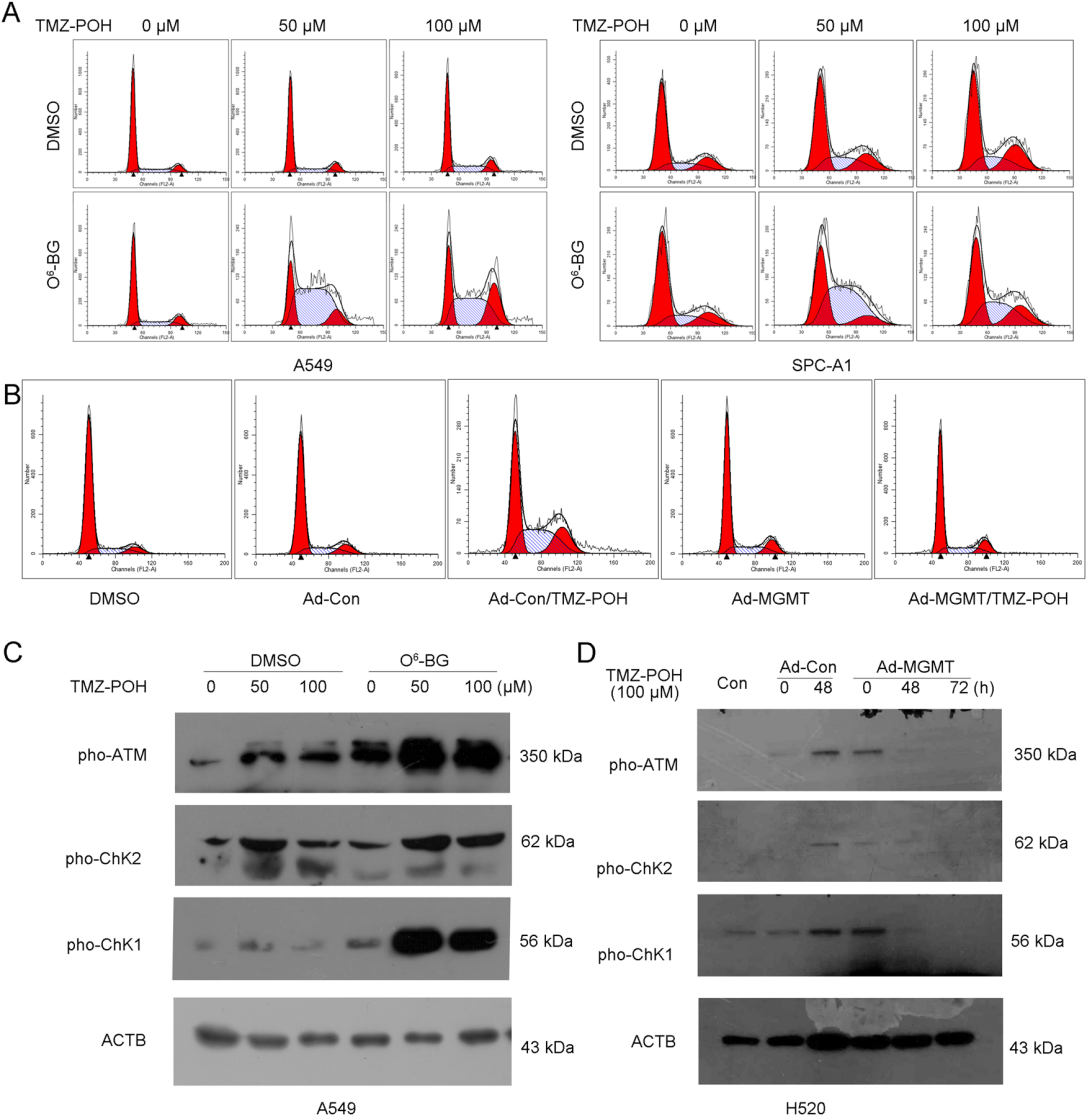
MGMT downregulation is required for cell cycle-dependent DNA damage induced by TMZ–POH. A549 and SPC-A1 cells were treated with 0, 50, 100 μM TMZ–POH for 48 h with or without O^6^-BG pretreatment, respectively; H520 cells was infected by Ad-MGMT or control for 24 h followed by 100 μM TMZ–POH treatment for 48 h. **a**,**b** The cell cycle distributions of A549, SPC-A1, and H520 cells treated with above drugs were analyzed. **c**,**d** Western blot analysis demonstrated p-ATM, p-ChK1, p-ChK2, and ACTB expression in above drug-treated A549 and H520 cells

Our previous study has reported TMZ–POH depends on its ability to induce cell cycle-dependent DNA damage^18,19^, so the influence of TMZ–POH-induced MGMT downregulation on G_2_/M arrest was also investigated. As shown in Fig. 3a, more population at the G_2_/M phase and less population at the G_1_ phase were detected significantly when treated with TMZ–POH, whereas combination of TMZ–POH and O^6^-BG led to more population accumulated at the S and G_2_M phases in A549 and SPC-A1 cells. These data were confirmed by MGMT overexpression as shown in Fig. 3b. TMZ–POH caused an obvious G_2_/M arrest in H520 cells, which was alleviated after Ad-MGMT infection, indicating TMZ–POH-induced cell cycle arrest was exacerbated by O^6^-BG-mediated MGMT inhibition, but alleviated by forced MGMT expression.

One of underlying mechanisms of TMZ–POH causes cell cycle arrest through activation of ATM-ChK1/2 signaling^18,19^. Our data supported this mechanism of TMZ–POH was also regulated by MGMT. Consistent with our previous study, TMZ–POH upregulated some DNA damage-related proteins, including phosphorylated ataxia telangiectasia mutated (ATM) and checkpoint kinases 1 and 2 (ChK1/2), and also all those proteins were elevated more significantly under MGMT inhibition by O^6^-BG in A549 cells (Fig. 3c), but were restored under Ad-MGMT infection in H520 cells (Fig. 3d), suggesting MGMT was involved in TMZ–POH-induced DNA damage. In addition, TMZ–POH-suppressed MGMT was also required for its role in CNE2 cells as shown in Figure S3, combination of O^6^-BG with TMZ–POH exhibited more potency than TMZ–POH alone in its cytotoxicity (Fig. S3A), apoptosis (Fig. S3B), cell cycle arrest (Fig. S3C), and DNA damage (Fig. S3D).

Our previous data has supported TMZ–POH-induced DNA damage due to ROS accumulation since it could be restored when ROS scavenged^18,19^. Hence, the ROS level was also evaluated, as shown in Figure S4A, combination of TMZ–POH and O^6^-BG led to an increase in ROS level compared with TMZ–POH alone. It is well-established that mitochondrial membrane potential collapse^28^ and PI3K/MAPK^29^ signaling works as a result from ROS accumulation. Our data also showed O^6^-BG-facilitated TMZ–POH-induced more mitochondrial membrane potential collapse (Fig. S4B) and activated PI3K/MAPK signaling (Fig. S4C).

### TMZ–POH downregulates MGMT independent of Wnt/β-catenin signaling

**Fig. 4:**
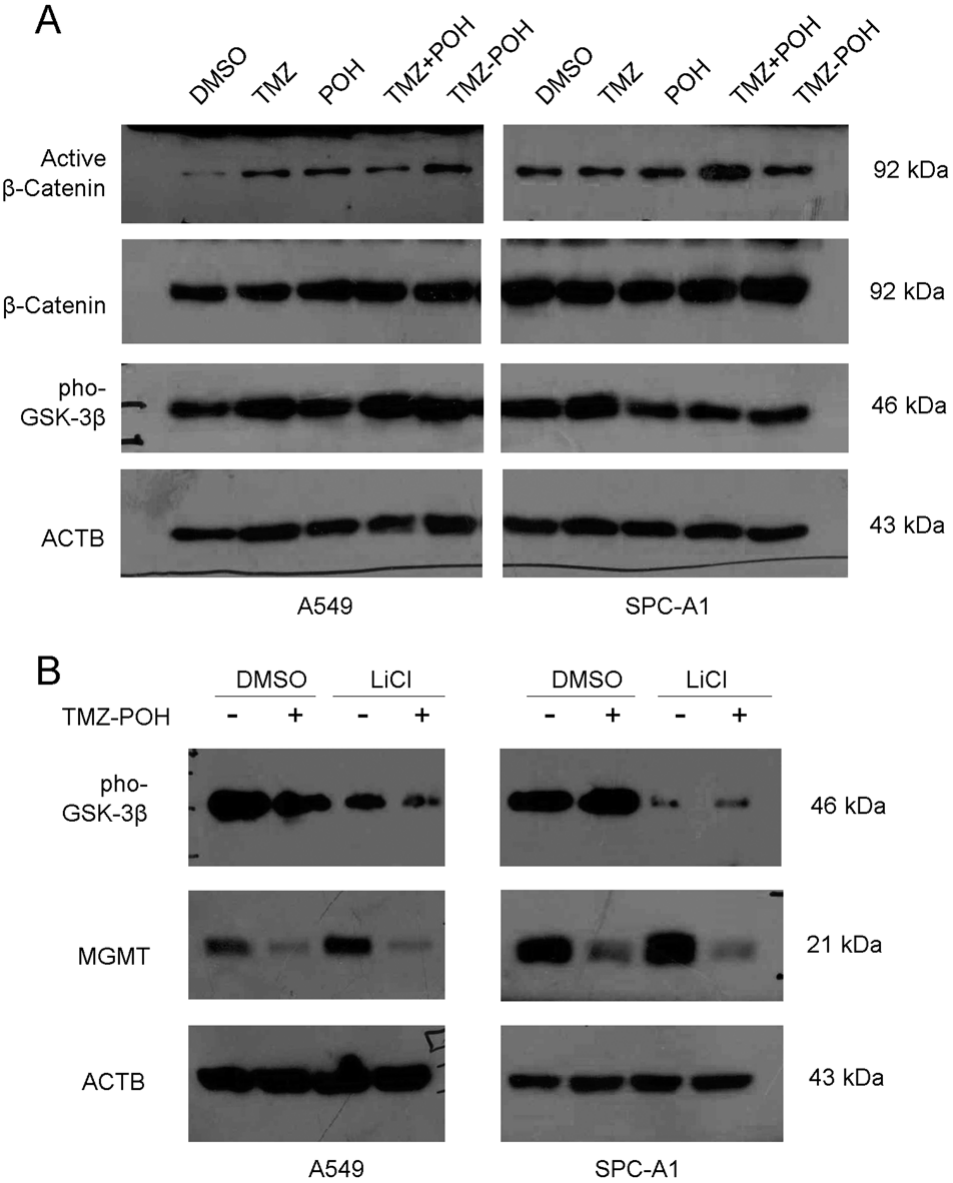
TMZ–POH downregulates MGMT independent of Wnt/β-catenin signaling. **a** A549 and SPC-A1 cells were treated with 100 μM TMZ, POH, TMZ + POH, TMZ–POH, or DMSO, respectively, for 48 h and subjected to western blot analysis to demonstrate active β-Catenin, β-Catenin, Pho-GSK-3β, ACTB protein level. **b** A549 and SPC-A1 cells were treated with 0, 100 μM TMZ–POH for 24 h with or without presence of 10 mM LiCl, and western blot analysis demonstrated pho-GSK-3β, MGMT, and ACTB expression

It has been reported that MGMT gene expression was regulated by Wnt/β-catenin signaling. Beta-catenin functions as a transcriptional co-activator, and directly regulates the expression of MGMT^7^. Therefore, whether Wnt/β-catenin signaling was involved in MGMT inhibition by TMZ–POH was evaluated. First, some components of Wnt/β-catenin signaling were detected including active form and inactive form β-catenin, phosphorylated glycogen synthase kinase-3β (GSK-3β), a negative regulator of Wnt pathway which can phosphorylate the amino terminal region of β-catenin, resulting in its ubiquitination and proteasomal degradation^30^. Unexpectedly, TMZ–POH seemed to cause no significant difference of the above protein level compared to its individuals alone or combination in A549 and SPC-A1 cells (Fig. 4a), indicating the absence of Wnt/β-catenin signaling in MGMT downregulation by TMZ–POH. Furthermore, lithium chloride (LiCl), an established inhibitor of GSK-3β, was administrated to activate Wnt/β-catenin^31^. As shown in Fig. 4b, although LiCl increased the expression of MGMT, it failed to restore MGMT inhibition by TMZ–POH in A549 and SPC-A1 cells. Collectively, these data suggest TMZ–POH downregulates MGMT independent of Wnt/β-catenin signaling.

### TMZ–POH downregulates MGMT in proteasomal degradation manner

**Fig. 5:**
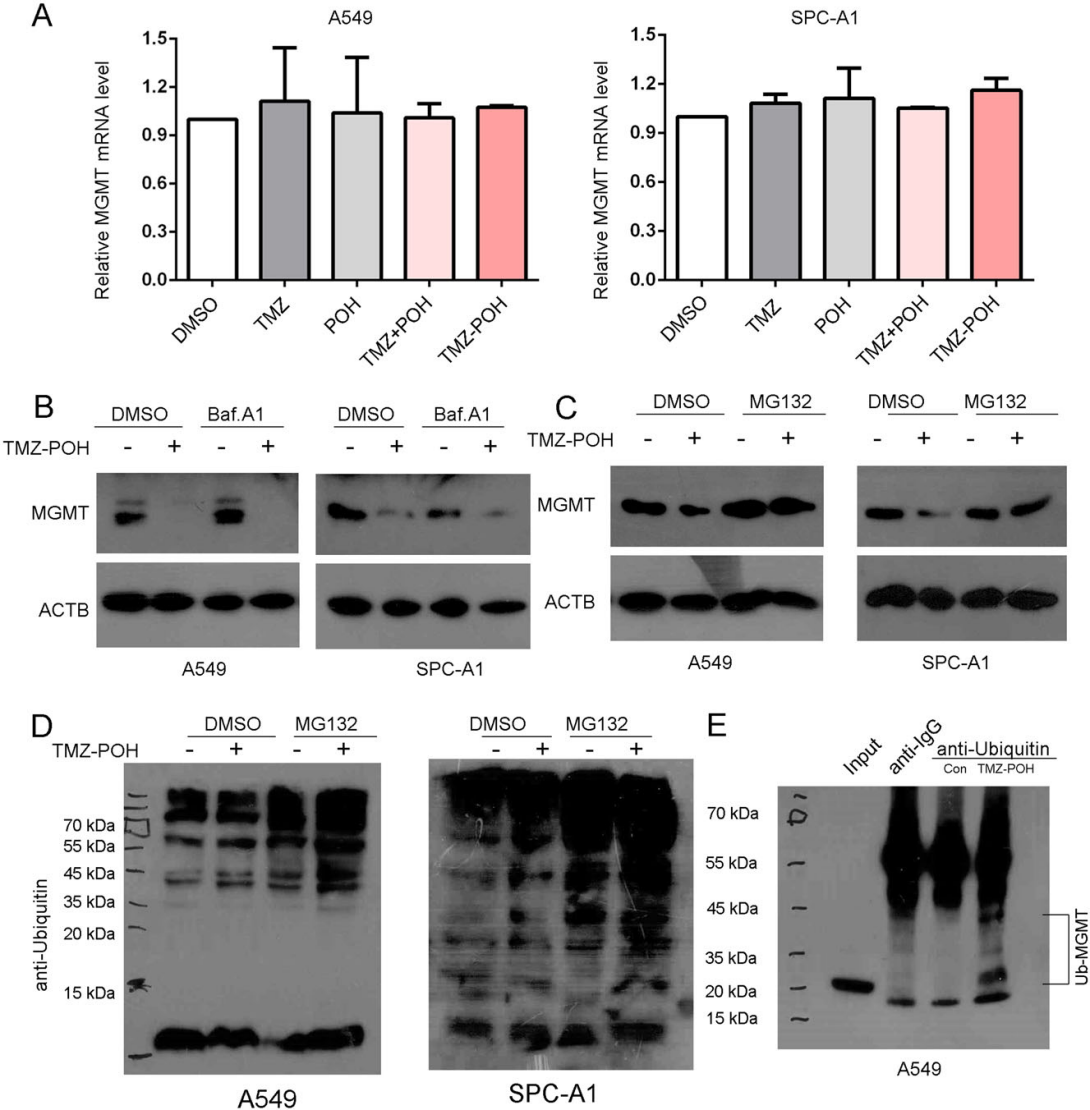
TMZ–POH downregulates MGMT in proteasomal degradation manner. **a** A549 and SPC-A1 cells were treated with 100 μM TMZ, POH, TMZ + POH, TMZ–POH, or DMSO, respectively, for 24 h, relative mRNA levels of MGMT were examined using specific primers. **b** A549 and SPC-A1 cells were treated with 0, 100 μM TMZ–POH for 24 h with or without pretreatment with Bafilomycin A1 (Baf.A1, 200 nM) and western blot analysis demonstrated MGMT and ACTB expression. **c**,**d** A549 and SPC-A1 cells were treated with TMZ–POH for 24 h with or without pretreatment with MG132 (10 μM), and lysates were subjected to western blot using antibody against MGMT (**c**) and Ubiquitin (**d**); or lysates were incubated with the antibody against Ubiquitin overnight for immunoprecipitation followed by western blot analysis using the antibody against MGMT (**e**)

To explore the underlying mechanisms by which TMZ–POH inhibited MGMT, the transcriptional level of MGMT was detected in A549 and SPC-A1 cells treated with TMZ–POH or its individuals alone or combination. The data showed no obvious difference of MGMT mRNA level when treated with TMZ–POH (Fig. 5a), suggesting MGMT downregulation by TMZ–POH did not attribute to MGMT’s translation but to its degradation.

Previous studies have demonstrated that the inactive MGMT is a short-lived protein, and suffers from degradation in mammals and yeast^23–25^. It is well-established that there are two pathways for protein degradation: one is the lysosomal pathway, the other is proteasomal. To identify the MGMT degradation manner, we pre-treated A549 and SPC-A1 cells with two protein degradation inhibitors, bafilomycin A1 (Baf. A1), an inhibitor of vacuolar proton pump (V-H^+^-ATPase) to block the lysosomal pathway^32^, MG132, a well-established 26S proteasome inhibitor. As shown in Fig. 5b, Baf. A1 failed to restore MGMT inhibition by TMZ–POH but MG132 succeeded (Fig. 5c), indicating TMZ–POH downregulated MGMT dependent on proteasomal but not lysosomal pathway. To further confirm this issue, the ubiquitination induced by TMZ–POH was also detected. As shown in Fig. 5d, TMZ–POH led to a higher level of ubiquitination in both A549 and SPC-A1 cells after pretreatment of MG132. Additional evidence for the involvement of the TMZ–POH in MGMT degradation was obtained by co-immunoprecipitation experiments, lysates from A549 cells was incubated with ubiquitin antibodies to pull down the proteins associated with ubiquitin. As shown in Fig. 5e, poly-ubiquitinated MGMT was observed in TMZ–POH-treated group. Taken together, these data reveal proteasomal degradation manner is involved in MGMT downregulation by TMZ–POH.

## Discussion

Despite the effectiveness of alkylating agents like TMZ in treating multiple types of malignancy, many patients still fail to benefit from this drug because of primary and acquired resistance, since MGMT highly expressing in about 80% of cancers^33^ can remove TMZ methyl adducts and ensues its resistance. In this study, we demonstrated that TMZ–POH, a newly designed TMZ analog, inhibited MGMT significantly in a concentration-dependent and time-dependent manner. Importantly, the inhibitory role of TMZ–POH in MGMT is a universal phenomenon observed in all detected cell lines, independent of the cell types, thus explaining the reason why TMZ–POH exerts its anti-tumor activity in cells with MGMT high expression even in TMZ-resistant gliomas. In addition, our data show that MGMT downregulation is required for TMZ–POH’s cytotoxicity, as evidence from TMZ–POH’s cytotoxicity enhanced by O^6^-BG but alleviated by MGMT overexpression, supporting the idea that MGMT is involved in the efficiency of alkylating agents. Therefore, our finding provides novel insights into the MGMT downregulation by TMZ–POH, and the role of MGMT downregulation in its cytotoxicity.

Importantly, our data showed that MGMT was recovered rapidly after withdrawing of TMZ–POH. The data suggest that TMZ–POH acted as a transient inhibitor of MGMT in contrast to O^6^-BG. The failure of O^6^-BG in clinical applications is mainly attributed to its bone marrow and hematologic toxicity, it can induce a persistent inhibition of MGMT systematically including hematopoietic system, whereas the longer MGMT suppression is likely to lead to a continued accumulation of alkylation DNA damage^14^. Although it remains to be evaluated, TMZ–POH will most likely avoid some of the hematological toxicities observed by systemic administration of small-molecule inhibitors of MGMT, since TMZ has less toxicity to the hematopoietic progenitor cells than other alkylating agents such as 1,3-bis(2-chloroethyl)-nitrosourea due to its failure in causing chemical cross-linking of the DNA strands^6^. Our observations suggest that TMZ–POH induced the short-term inhibition of MGMT is likely beneficial in rescuing the host tissues from continued genomic injury, and free from at least partially hematologic toxicity.

MGMT, a suicide enzyme that removes O^6^-meG to its unchanged state in a one-step but renders MGMT inactive, is regulated by multiple mechanisms including epigenetic silence by its promoter methylation and transcriptional regulation by Wnt/β-catenin signaling as a direct target of β-catenin^7^. Unexpectedly, Wnt/β-catenin showed less influence on MGMT inhibition by TMZ–POH, and the GSK-3β inhibitor LiCl failed to restore MGMT expression which was inactivated by TMZ–POH. Interestingly, several lines of evidence indicate that TMZ–POH inhibits MGMT in a proteasomal degradation pathway. First, TMZ–POH did not alter MGMT mRNA level, and a transcriptional inhibitor ActoD didn’t induce an obvious decrease of MGMT expression, suggesting inhibition of MGMT by TMZ–POH didn’t attribute to MGMT’s generation but to its degradation. Second, previous studies have demonstrated MGMT undergoes degradation in mammals and yeast^23,24^. Lysosomal inhibitor Baf. A1 failed to restore MGMT inhibition by TMZ–POH but 26S proteasomal inhibitor MG132 succeeded, indicating TMZ–POH downregulates MGMT dependent on proteasome. Finally, TMZ–POH led to a higher level of ubiquitination, and poly-ubiquitinated MGMT was observed when treated with TMZ–POH coincident with the previous result that MGMT are degraded via ubiquitination-dependent proteolysis^23^. It has been reported MGMT acts as a target for degradation by a complex of the viral E6 protein and E6AP in mammals^24^ as well as a physiological substrate of both the Ubr1-dependent N-end rule and UFD pathway in yeast^25^. Whether above pathways are also involved in MGMT degradation by TMZ–POH needs more exploration. Taken together, the current study supports TMZ–POH degrades MGMT dependent on ubiquitination proteolysis.

In summary, in this paper we explore the role and mechanisms of TMZ–POH in MGMT downregulation, and those of MGMT downregulation in TMZ–POH’s cytotoxicity, thus proposing TMZ–POH as a potential therapeutic candidate of cancers.

## Methods and materials

### Cell lines and chemicals

Human NSCLC-derived cell lines A549, SPC-A1, NCI-H460, and NCI-H520 were purchased from American Type Culture Collection (Manassas, VA, USA) and China Center for Type Culture Collection (Wuhan, China). All these cells were grown in Dulbecco’s modified Eagle’s medium (DMEM, Gibco, Invitrogen, Carlsbad, CA, USA) supplemented with 10% 10% fetal bovine serum (Gibco, Invitrogen) and antibiotics (penicillin/streptomycin, 100 U/ml) at 37 °C in 5% CO_2_.

TMZ–POH and POH were provided by Neonc Technologies, Inc. (Los Angeles, USA) and diluted with DMSO to make stock solutions of 100 mM. TMZ, O^6^-benzylguanine (O^6^-BG), MG132, baflomycin A1 (Baf. A1) (all from Sigma-Aldrich) were dissolved in DMSO. In all cases of cell treatment, the final DMSO concentration in the culture medium never exceeded 0.5%. LiCl (Amresco, Solon, OH, United States) was dissolved in H_2_O. Stock solutions of all drugs were stored at –20 °C.

### Adenovirus infection

Recombinant adenoviral vector carrying the human MGMT gene (Ad-MGMT) was purchased from Vigene Bioscience (Shandong, China). H520 cells were plated in 6-well plates at a density of 1 × 10^5^ cells per well. Cells were infected at an MOI of 2 with Ad-Empty or Ad-MGMT the next day for 24 h. After washing with PBS twice, cells were treated with TMZ–POH for the next 24, 48, 72 h, respectively.

### Cell viability assay

Cells were plated in 96-well plates at 3000–4000 per well dependent on cell type. After drug treatment for 48 h, MTT assay was done through the addition of 3-(4,5-dimethylthiazol-2-yl)-2,5-diphenyltetrazolium bromide) solution (made by adding 5 mg/ml in PBS) at 10 μl per well, followed by incubation at 37 °C in 5% CO_2_ for 4 h. Formazan crystals that formed were solubilized with 100 μl of acidified (0.01 M HCl) 10% SDS (sodium dodecyl sulfate) overnight at 37 °C. Absorbance (*A*) at 570 nm (reference wavelength: 630 nm) was read on a Bio-Rad 680 microplate reader (Bio-rad 680, Bio-Rad Laboratories, Hercules, USA). The cell viability was calculated with the following formula: cell viability = *A*^drug-treated^/*A*^DMSO^ × 100%. The experiment was repeated three times.

### Detection of apoptotic cells

Apoptosis was evaluated by using the Annexin V-FITC Apoptosis Detection Kit (BD Biosciences Pharmingen, San Diego, USA) according to the description provided by the manufacturer. After drug treatment, the cells were trypsinized, collected, and stained with FITC-Annexin V & propidium iodide (PI) for 15 min in the dark. The stained cell population were determined using by a FACS Calibur instrument (Becton Dickinson, USA) and the data were analyzed using FlowJo Software 7.6 (Treestar, Inc., San Carlos, CA). Three independent experiments were carried out.

### Cell cycle analysis

Cells growing in 12-well plates were treated by above agents, cells were collected and washed once with PBS, and then re-suspended and fixed in 70% ethanol overnight. After incubation in 1 ml of PI staining solution (0.1% Triton X-100, 200 μg/ml DNase-free RNase A, 20 μg/ml PI) for 1 h at room temperature, DNA content was evaluated by a FACS Calibur instrument (Becton Dickinson, Bedford, MA, USA) and the distribution of cell cycle phases were determined using ModiFit software (Topsham, ME, USA). Three independent experiments were carried out

### Determination of ROS accumulation

ROS accumulation in cells following the above treatment were evaluated using 2′, 7′-dichlorofluorescein diacetate (DCFH-DA) kit (Beyotime, China) according to the manufacturer’s protocol. Briefly, after indicated treatment for 48 h, the cancer cells were washed with serum-free DMEM twice and incubated with DCF-DA (20 µM, diluted in serum-free DMED) for 20 min at 37 °C in 5% CO_2_, and then collected and suspended in PBS. The florescence intensity of dichlorofluorescein was measured by a FACS Calibur instrument (Becton Dickinson, USA) with the excitation source at 488 nm and emission at 525 nm and the data were analyzed using FlowJo Software 7.6 (Treestar, Inc., CA). Three independent experiments were carried out.

### Analysis of mitochondrial transmembrane potential (Δψm)

Cancer cells grown in six-well plates overnight were exposed to indicated drugs, then stained with the cationic dye 5,5′,6,6′-Tetrachloro-1,1′,3,3′-tetraethyl-imidacarbocyanine iodide (JC-1; Beyotime, China) to demonstrate the state of mitochondrial transmembrane potential according to the manufacturer’s protocol. Briefly, cells were harvested and transferred to 1.5 ml tubes, and then incubated with JC-1 (5 μg/ml) in a 37 °C incubator for 20 minutes after washing twice with PBS. Subsequently, cells were collected and subjected to flow cytometry (Becton Dickinson, USA) to detect the change of JC-1 florescence. The data were analyzed using BD FACS DIVA software (Becton Dickinson, USA). Two independent experiments were carried out.

### RNA isolation and real-time PCR

Total RNA was extracted from cells using Trizol Reagent (Invitrogen). Real-time PCR was performed using UltraSYBR Mixture (CWBIO, CW0956) and specific primer pairs. The sequences of the sense and antisense primers were as follows: MGMT: 5′-CTCTTCACCATCCCGTTT-3′; 5′-AATCACTTCTCCGAATTTCAC-3′; ACTB: 5′-TTAGTTGCGTTACACCCTTTC-3′; and 5′-GCTGTCACCTTCACCGTTC-3′. Using the 2^ΔΔCt^ method, our data were reported as the fold change in experimental group normalized to an endogenous reference gene (ACTB) and relative to control group.

### Co-immunoprecipitation

Cancer cells grown in 100 mm dishes overnight at a density of 1 × 10^6^ cells per dish were exposed to MG132 for 4 h, followed by treatment with 100 μM TMZ–POH for another 24 h. Then cells were collected and lysed with cold Cell Lysis Buffer (Beyotime, Beijing, China), and incubated with antibody against Ubiquitin or isotype IgG (2 μg, Proteintech, Wuhan, China) overnight at 4 °C with gentle rotation. 40 μl Protein A + G Agarose (Santa Cruz Biotechnology, Inc, Dallas, USA.) was added to per tubes and rotated at 4 °C for 3 h. Beads were precipitated by centrifugation at 16,000 × *g* for 30 s and washed five times with cold Cell Lysis Buffer. The pellets were re-suspended in 1 × SDS-PAGE Sample Loading Buffer (Beyotime, Beijing, China), and incubated at 100 °C for 5 min. The supernatants were used for western blot analysis using the antibody against MGMT.

### Western blots

Cells were lysed in cell lysis buffer (20 mM Tris pH 7.5, 150 mM NaCl, 1% Triton X-100) (Beyotime, Beijing, China) supplemented with 0.5 mM phenylmethanesulfonyl fluoride (Beyotime), and the total cellular protein concentration was determined with a BCA Protein Assay Kit (Thermo Fisher Scientific Inc., Rockford, USA). A total of 50 μg of protein was separated on SDS-PAGE and transferred onto PVDF membranes (Millipore, Billerica, MA, USA). Membranes were then blocked with 5% evaporated skimmed milk (Bio-rad, USA) in Tris-buffered saline (50 mM Tris-HCl, pH 7.5, 150 mM NaCl) containing 0.1% Tween-20 for 1 h, and probed overnight at 4 °C with the following primary antibodies: antibodies against human MGMT, phospho-ATM, phospho-CHEK1/2, phospho-H2AX, β-Catenin, active β-Catenin, phospho-GSK-3β (all 1:1000; Cell Signaling Technology, Danvers, MA, USA), antibody against Ubiquitin (Proteintech, China), antibody against ACTB (1:2000; Zsbio, Beijing, China), followed by incubation with horseradish peroxidase coupled secondary anti-mouse or anti-rabbit antibodies (Zsbio, China) for 1 h at room temperature. The protein bands were visualized using ECL-blotting detection reagents (Bio-rad, USA), and developed and fixed onto X-ray films. ACTB was served as a loading control.

### Statistical analysis

Statistical significance was evaluated with data from at least three independent experiments. GraphPad Prism 6.02 (GraphPad Software, San Diego, CA, USA) was used for data analysis. Statistical analysis was carried out using Student's *t* test. Data are presented as the mean ± SEM. For all statistical tests, significance was established at *p* < 0.05.

## Electronic supplementary material

Figure S1

Figure S2

Figure S3

Figure S4

Figure Legends
