# Supplementary figures and images for: Temozolomide–perillyl alcohol conjugate downregulates O6-methylguanin DNA methltransferase via inducing ubiquitination-dependent proteolysis in non-small cell lung cancer
Source: Cell Death Dis. 2018 Feb 9;9(2):202. doi: 10.1038/s41419-017-0193-2 (PMC5833843; doi:10.1038/s41419-017-0193-2)

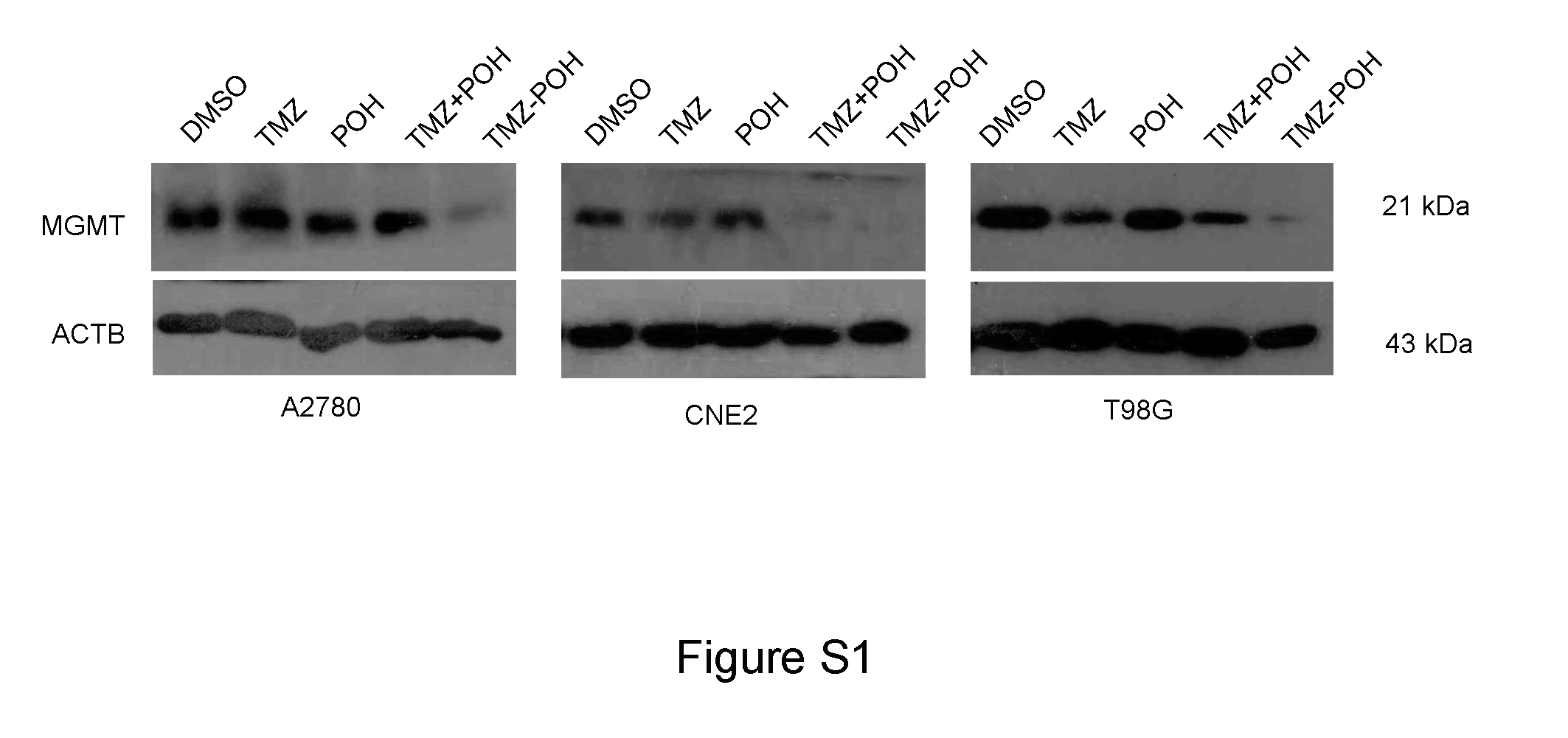

Supplement: Supplementary file 1 — Figure S1 [file 41419_2017_193_MOESM1_ESM.jpg]

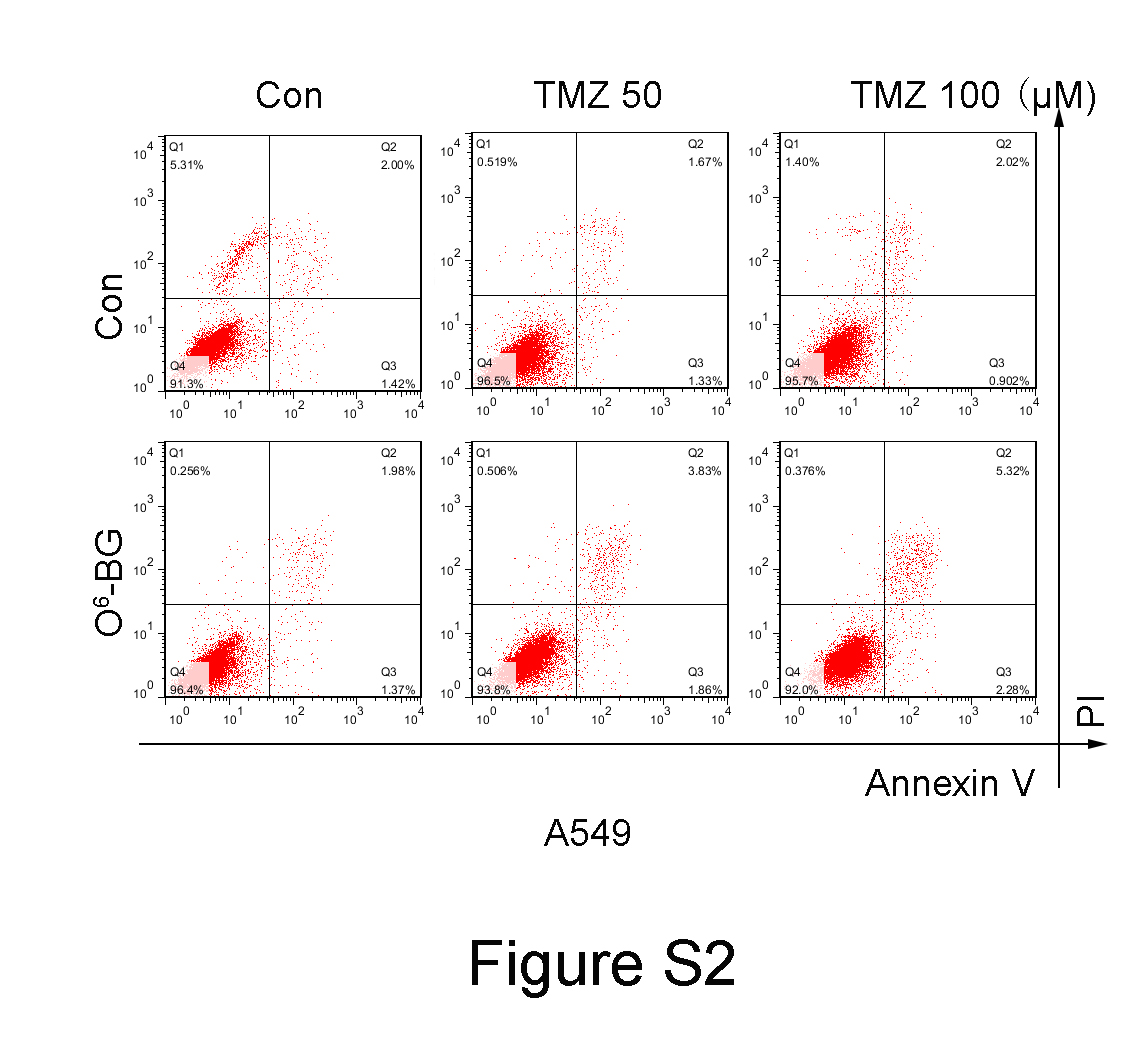

Supplement: Supplementary file 2 — Figure S2 [file 41419_2017_193_MOESM2_ESM.jpg]

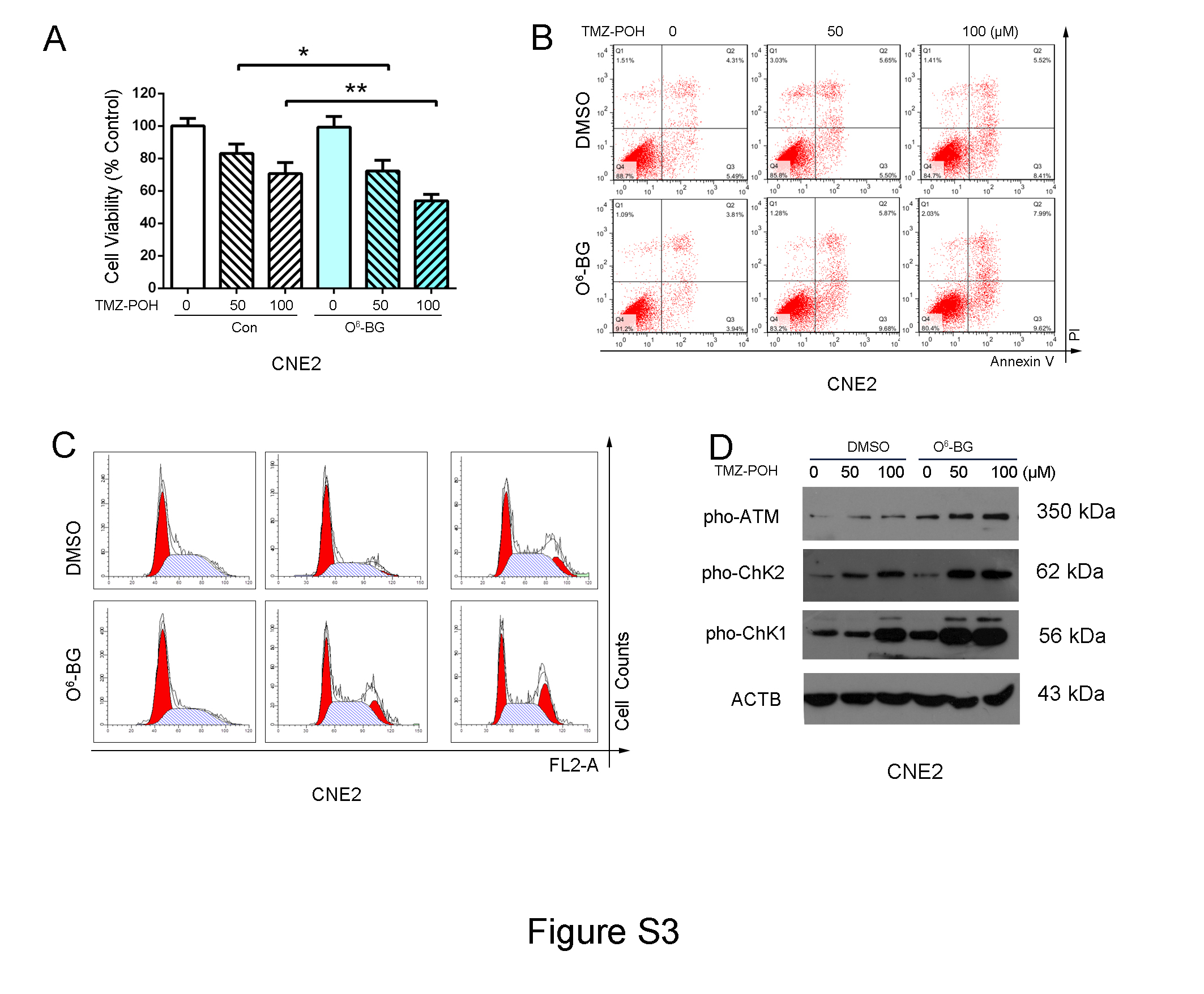

Supplement: Supplementary file 3 — Figure S3 [file 41419_2017_193_MOESM3_ESM.jpg]

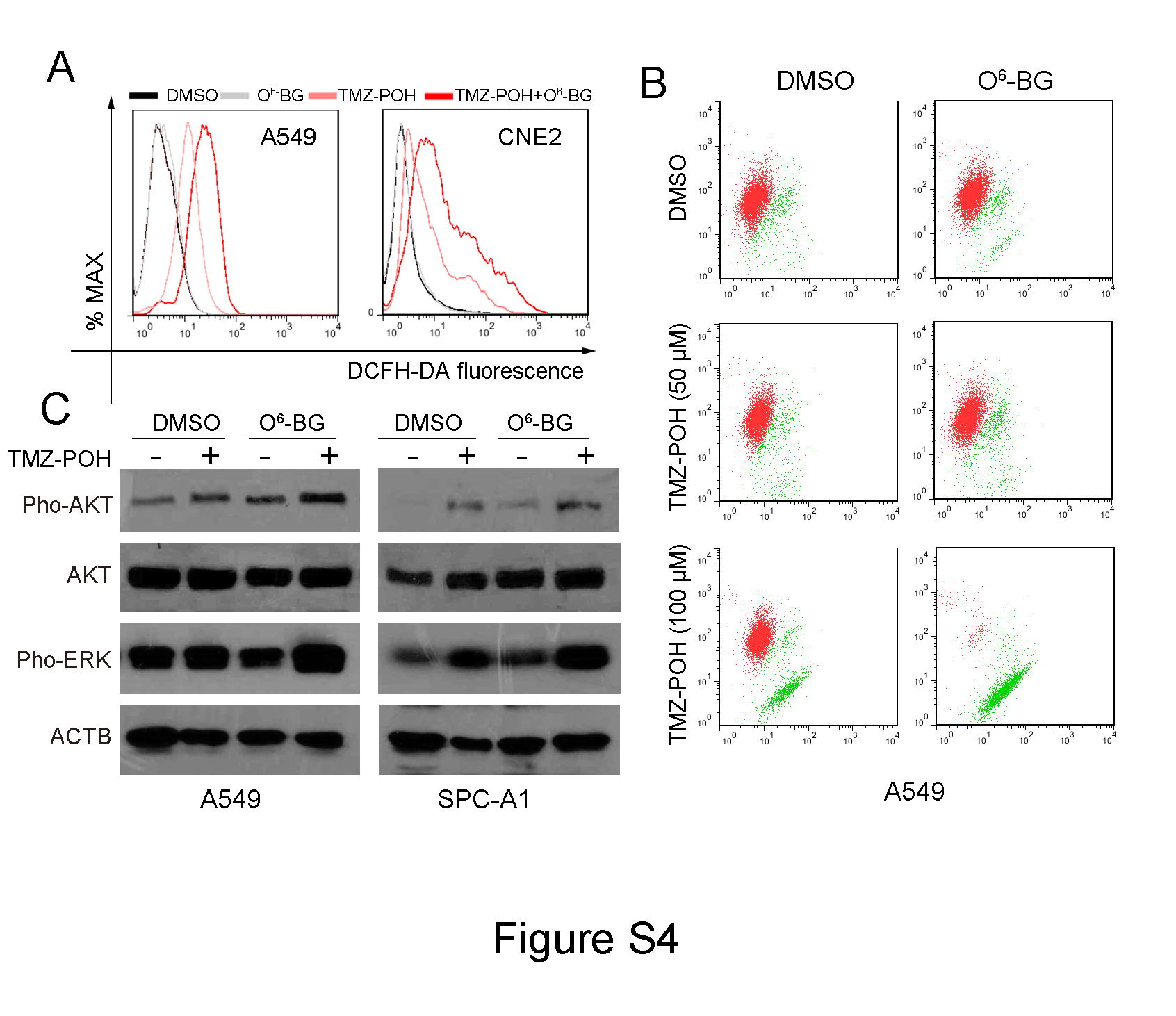

Supplement: Supplementary file 4 — Figure S4 [file 41419_2017_193_MOESM4_ESM.jpg]
