# Supplementary material for: Temozolomide–perillyl alcohol conjugate downregulates O6-methylguanin DNA methltransferase via inducing ubiquitination-dependent proteolysis in non-small cell lung cancer
Source: Cell Death Dis. 2018 Feb 9;9(2):202. doi: 10.1038/s41419-017-0193-2 (PMC5833843; doi:10.1038/s41419-017-0193-2)
Supplement: Supplementary file 5 — Figure Legends [file 41419_2017_193_MOESM5_ESM.docx]

SUPPLEMENTARY FIGURE LEGENDS

Figure S1: TMZ-POH downregulates MGMT protein. MGMT protein level was evaluated by western blots in CNE2, A2780 and T98G cells; ACTB acts as control.

Figure S2: O^6^-BG facilitates TMZ induced apoptosis in A549 cells. A549 cells were treated with 0, 50, 100 μM TMZ for 48 h with or without presence of 25 μM O^6^-BG respectively, and then subjected to apoptosis assay.

Figure S3: MGMT downregulation is required for TMZ-POH’s potency in CNE2 cells. CNE2 cells were treated with 0, 50, 100 μM TMZ-POH for 48 h with or without presence of 25 μM O^6^-BG respectively, and then subjected to MTT assay (A), apoptosis assay (B), cell cycle detection (C) and DNA damage related proteins analysis (D). The results shown are means ±SD; *p < 0.05; **p < 0.01.

Figure S4: (A) A549 and CNE2 cells were treated with 100 μM TMZ-POH with or without O^6^-BG pre-treatment respectively, intracellular ROS levels were measured using the fluorescent probe DCFH-DA. (B) The above drug treated A549 cells were detected using JC-1 flow cytometry. (C) Lysates from above drug-treated A549 and SPC-A1 cells were subjected to western blot to detect pho-AKT, AKT, pho-ERK and ACTB.
